# Suprapubic Cystotomy for Stone; Some Complications

**Published:** 1897-09

**Authors:** Virginius W. Harrison

**Affiliations:** Richmond, Va., Lecturer on Surgery, University College of Medicine, Richmond, Va.


For the Texas Medical Journal.

### SUPRAPUBIC CYSTOTOMY FOR STONE; SOME COMPLICATIONS.

BY VIRGINIUS W. HARRISON, A. M., M. D., RICHMOND, VA.,
Lecturer on Surgery, University College of Medicine, Richmond, Va.

[Read before the Richmond Academy of Medicine and Surgery, July 15, 1897.]

ON MAY 22,1897, I did a suprapubic cystotomy for stone on Paul A., white, age 16. After cutting through the abdominal parietes, I found the peritoneum presenting itself in front of the bladder, almost touching the pubic bone. It was some time before I was certain that the peritoneum was far enough out of the field of operation to make the incision into the bladder without doing harm to it, but this was finally accomplished, and without further trouble, a small mulberry calculus was removed. A catheter was placed in the bladder, and iodoform gauze loosely packed around it to favor rapid and thorough drainage; and a stitch was taken in the upper angle of the wound, extra gauze being placed here to protect the peritoneum as well as possible. The patient was put to bed in good condition.

The usual preparatory treatment was administered; the bladder being washed out just before the operation, and filled with sterilized water, and the rectal bag was used.

The patient did well until 6 a. m., when he became restless and suffered pain in the abdomen; pulse, 18; temperature, 101.4°. At 12 m. pulse, 110; temperature, 103.2°, with all the symptoms of septic infection of the peritoneal cavity, viz.: Distended and rigid abdominal muscles; face flushed and anxious, nausea, etc. It was determined to open the abdomen immediately. The bladder was first washed out, and the incision which had been made in the bladder at the first operation was closed with interrupted silk sutures; a catheter was placed in the urethra to insure drainage and to prevent the urine from soiling the peritoneum and wound after it had been washed and dressed.

On opening the abdominal cavity, the visceral and parietal pelvic peritoneum was found inflamed and bathed in a dirty sero-purulent fluid, an evidence of fibrino-purulent peritonitis. Near the bladder, the peritoneum looked dark and very ugly, and in a few hours, without irrigation and drainage, would haye been attended by diffuse suppuration or septic peritonitis. The cavity was irrigated for some time with hot sterilized water. The lower end of the peritoneal wound was closed with a continuous silk suture; the cavity well drained with iodoform gauze, and silk-worm sutures placed in such a position as to close the abdominal wound after removal of the gauze. The patient was put to bed in a fairly good condition; pulse, 120; temperature, 101.2°. He rallied well, with a steady decrease of temperature, until May 26, at 2:30 p. m. The drainage had ceased, the gauze was removed, and the abdominal wound closed; temperature, 100.60°; pulse, 88; respiration, 26. By 7:30 p. m., temperature, 102°; pulse, 89; respiration, 26. A dose of salts was given, and at 9 p. m. an enema was admininterede with good effect, reducing the temperature to 100.8° by 10 p. m.

As he had been much nauseated, the patient was fed and stimulated by enemas. The bladder commenced to drain through the suprapubic wound on the sixth day after operation, showing that the sutures in the bladder had given way. The catheter was removed from the urethra. The abdominal wound soon became infected and sloughed somewhat. The abdominal stitches were removed on June 2nd, and the wound dressed with chloral solution. From this time the temperature varied from 98.8° to 99.6° until June 17, when, at 6 a. m.- the condition was, temperature, 101°; pulse, 102; respiration, 24. At 5 p. m., temperature, 103,0°; pulse, 116; respiration, 26; delirious, and in a condition of profound sepsis. The origin was difficult of location. When not delirious, he would complain of great pain in the head, back and abdomen, and of nausea. Refusing all nourishment, he was fed by nutritive enemas, and also was given one grain of calomel every hour until six grains had been taken; then a full high enema was given which proved very effective.

June 18, 9:15 a. m., temperature, 100°; pulse, 105°; respiration, 24°; complained of feeling chilly, still nauseated and suffering pain in his head and abdomen; 5 p. m., temperature, 103°; pulse, 100; respiration, 26. This condition continued, with morning temperature about 100° and afternoon temperature 103°, until June 21st, at 3:30 p. m., when he became very restless and weak, suffered intense pain in his back, in the region of the right kidney, aching in his limbs, head and abdomen; pulse, 120; temperature, 104°; respiration, 34°. Sponge baths of iced water and alcohol were ordered to be given every two hours, and the nurse was told to push stimulants and nourishment. The next morning, at 8:30, a great deal of pus escaped from the suprapubic wound and continued for several days. Whenever an enema was given to wash out the bowel, he would pass urine through the urethra, which would be filled with pus. Temperature remained up until June 23, 4 a. m., when his condition improved. He looked and felt better; temperature, 100°; pulse, 84; respiration, 22. June 25th, he again suffered from absorption of pus, temperature going up to 103.4°. The wound was washed out, as had been done before with peroxide of hydrogen, every six hours. By the 28th the temperature was normal, but would vary during each day, going as high as 100°.

The poor little fellow's troubles had not yet ceased, for on July 7th he had another rise of temperature, it being at 6 p. m., 103.8°, with all symptoms of sepsis. The wound was draining well and freely, so another focus of infection had to be sought. Later in the evening he passed a large quantity of decomposed mucus from the rectum, and continued to do so three or four times daily for several days, the temperature varying during this time from 101.5° to 103.8°. The trouble in the rectum was due, no doubt, to the effect of the nutrient enemata he had been having so continuously during his illness. After washing out the bowel for several days, the temperature was again brought down to normal by the 10th, remaining so until July 14th, when it again rose to 102.6°, from the same cause in the bowel. After washing out the latter for a few days, it fell to normal and remained so. To-day he was removed to his home in this city, with the abdominal wound nearly healed, water being passed *per via naturalis*, and in an apparently good condition after nine weeks' illness.

Several interesting points might be brought out in reviewing this, but, in concluding, I will only refer to two: 1. Suppurative septic peritonitis may follow suprapubic cystotomy without mechanical injury to the peritoneum. 2. The early, prompt, and thorough flushing of the abdominal cavity, followed by drainage for suppurative septic peritonitis will save life, where a few hours' delay would only bring surgery into disrepute; and the patient's life would be doomed.

